# Neuroendocrine regulation of fat metabolism by autophagy gene *atg‐18* in *C. elegans* dauer larvae

**DOI:** 10.1002/2211-5463.12708

**Published:** 2019-08-12

**Authors:** Ray Jia, Jiuli Zhang, Kailiang Jia

**Affiliations:** ^1^ Department of Biological Sciences Florida Atlantic University Jupiter FL USA

**Keywords:** *atg‐18*, autophagy, fat metabolism, *daf‐2*, insulin, dauer

## Abstract

In environments with limited food and high population density, *Caenorhabditis elegans* larvae may enter the dauer stage, in which metabolism is shifted to fat accumulation to allow larvae to survive for months without food. Mutations in the insulin‐like receptor gene *daf‐2* force *C. elegans* to constitutively form dauer larva at higher temperature. It has been reported that autophagy is required for fat accumulation in *daf‐2* dauer larva. However, the mechanism underlying this process remains unknown. Here, we report that autophagy gene *atg‐18* acts in a cell nonautonomous manner in neurons and intestinal cells to mediate the influence of *daf‐2* signaling on fat metabolism. Moreover, ATG‐18 in chemosensory neurons plays a vital role in this metabolic process. Finally, we report that neuronal ATG‐18 functions through neurotransmitters to control fat storage in *daf‐2* dauers, which suggests an essential role of autophagy in the neuroendocrine regulation of fat metabolism by insulin‐like signaling.

**Abbreviations**
IGF: insulin‐like growth factor
NGM: nematode growth media

Living organisms accumulate fat as an energy resource to prevent food deprivation. In the presence of food, *Caenorhabditis elegans* goes through four larval stages and develops to a fertile adult. However, in an environment with limited food and high population density, *C. elegans* larvae may arrest development during the second molt and enter the dauer stage 1. The dauer larva is a dispersal stage that is stable for months under adverse environmental conditions and is an example of facultative diapause 1. A dauer larva is radially shrunken with a constricted intestine, closed buccal cavity, and specialized cuticle morphology and is resistant to detergents such as sodium dodecyl sulfate. Dauer larvae store lipids in intestinal and hypodermal cells and can survive for months without feeding 2.

Molecular studies of dauer mutants have revealed that three functionally overlapping neural pathways, including the insulin‐like growth factor (IGF) 3, 4, transforming growth factor‐β 5, 6, and the cyclic guanosine monophosphate 7 pathways, control dauer formation in response to dauer‐inducing environmental cues. DAF‐2, an insulin/IGF receptor, regulates fat metabolism and dauer morphogenesis by inhibiting the activity of DAF‐16, a member of the forkhead family of transcription factors 3, 8, 9. The *daf‐2* pathway also regulates adult lifespan, and mutations in *daf‐2* increase lifespan 10. DAF‐2 was reported to work in both neurons and intestinal cells, while DAF‐16 acts mainly in the intestine to control *C. elegans* lifespan 11, 12, 13. It has been shown that mutations of *bec‐1*, the worm ortholog of autophagy gene *atg6*, suppress fat accumulation, dauer morphogenesis, and the extended lifespan of *daf‐2* mutants 14.

Autophagy is an evolutionarily conserved lysosomal degradation pathway that promotes degradation of cytosolic components. Macroautophagy (hereafter referred to as autophagy) shuttles cytosolic components to lysosomes using a double membrane‐bound vesicle called an autophagosome 15. The fusion of the lysosomes and the autophagosome results in an autolysosome, the inside of which contains lysosomal hydrolases that proceed to hydrolyze the shuttled cytosolic components. The new carbohydrates, amino acids, nucleosides, and fatty acids produced by the degradation of cytosolic components can be used by the cell to maintain cellular metabolism 15. Studies have shown that the inhibition of autophagy leads to decreased lipid accumulation in *C. elegans daf‐2* mutant dauer larva 14.

Autophagy gene *atg‐18* encodes a protein that belongs to the WD repeat protein interacting with phosphoinositides protein family 16. We recently reported that mutations in the *atg‐18* gene can suppress autophagy induction in *daf‐2* mutants, and tissue‐specific expression of *atg‐18* can restore autophagy activity in corresponding tissues of *daf‐2*;*atg‐18* mutants 17. Here, we examined the tissue‐specific requirement of *atg‐18* for fat accumulation in *daf‐2* mutant dauer larvae. Our results suggest that autophagy in chemosensory neurons and intestinal cells plays an important role in DAF‐2‐regulated fat metabolism in *C. elegans* dauer larva.

## Materials and methods

### Strains and culture conditions

All strains were grown on nematode growth media (NGM) agar plates seeded with *Escherichia coli* strain OP50 and maintained at 15 °C as described by Brenner 18. The NGM agar plates were prepared according to a standard procedure 19. All the chemicals were purchased from the Fisher Scientific except the agar (CRITERION™ Agar; Hardy Diagnostics, Santa Maria, CA, USA) provided by VWR (catalog #89405‐068) and the cholesterol provided by Sigma (catalog #C8667, St. Louis, MO, USA). The following two strains were purchased from the Caenorhabditis Genetics Center (CGC): VC893 *atg‐18*(*gk378*, Minneapolis, MN, USA) and CB246 *unc‐64*(*e246*). *daf‐2*(*e1370*) and *E. coli* strain OP50 are gifts from Donald Riddle. All mutants are derived from the wild‐type Bristol N2 strain. Construction of *daf‐2*(*e1370*);*atg‐18*(*gk378*), *daf‐2unc‐64*(*e246*);*atg‐18*, and all extrachromosomal (*Ex*) array transgenic lines has been reported recently 17. Genotypes of transgenic lines used are as follows: *daf‐2*;*atg‐18*;* Ex*[*Patg‐18*::*atg‐18 + rol‐6*(*su1006*)], *daf‐2*;*atg‐18*;* Ex*[*Punc‐119*::*atg‐18 + rol‐6*(*su1006*)], *daf‐2*;*atg‐18*;* Ex*[*Pges‐1*::*atg‐18 + rol‐6*(*su1006*)], *daf‐2*;*atg‐18*;* Ex*[*Pdpy‐7*::*atg‐18 + rol‐6*(*su1006*)], *daf‐2*;*atg‐18*;* Ex*[*Pmyo‐3*::*atg‐18 + rol‐6*(*su1006*)], *daf‐2*;*atg‐18*;* Ex*[*Pgpa‐3*::*atg‐18 + rol‐6*(*su1006*)], *daf‐2*;*atg‐18*;* Ex*[*Pdaf‐11*::*atg‐18 + rol‐6*(*su1006*)], *daf‐2*;*atg‐18*;* Ex*[*Punc‐42::atg‐18 + rol‐6*(*su1006*)]*,* and *daf‐2*;*atg‐18*;* Ex*[*Podr‐2::atg‐18 + rol‐6*(*su1006*)]. The tissue‐specific promoters are as follows: *Pges‐1* for the intestinal cells 12; *Punc‐119* for all neurons 12; *Pdpy‐7* for hypodermal cells 20; *Pmyo‐3* for body wall muscle cells 21; *Pdaf‐11* in ASE, ASI, ASJ, ASK, AWB, and AWC 7; *Punc‐42* in ASH 22; *Pgpa‐3* in ADF, ADL, ASE, ASG, ASH, ASI, ASJ, ASK, AWA, and AWC 23, 24; and *Podr‐2* in ASG 25. Some amphid neuron‐specific promoters are also active in other nonamphid neurons that are not listed here.

### Fat staining

#### Sudan black B staining

*daf‐2*(*e1370*);*atg‐18*(*gk378*) mutants are lethal at 25 and 20 °C. The animals arrest development at egg and L1 larval stages. Therefore, all strains were grown at 15 °C. L4 hermaphrodites were picked up and allowed to develop at 15 °C for 24 h. The 1‐day‐old adults were transferred to fresh food plates and allowed to lay eggs at 15 °C for 16 h. The adults were removed, and eggs/L1s were shifted to 25 °C and incubated for 3 days (72 h). The dauer animals were picked up for staining. At least one hundred dauer larvae for each strain were picked up for Sudan Black B staining. For N2 and *atg‐18*(*gk378*) animals, L3‐stage larvae that were comparable to dauer larvae were used for staining. Collected animals were washed two to three times with M9 buffer. Paraformaldehyde stock solution (10%) was added to a final concentration of 1%. The samples were frozen in dry ice/ethanol and then thawed under a stream of warm water. After a total of three freeze–thaw cycles, the worms were dehydrated through ethanol solutions and then stained with Sudan Black B as described by Kimura *et al*. 3. After staining, all animals were examined for fat accumulation. The stained worms were mounted on a 2% agarose pad and observed under a Zeiss upright fluorescence microscope (Axio Imager A2, Zeiss, Oberkochen, Germany). To compare the fat content in different strains, the pictures were taken with the same camera setting using the Zeiss AxioCam ICm1 digital camera at 1000× magnification. The DIC filter and the Zeiss AxioVision 4.8 were used for imaging.

#### Nile red staining

Nile red staining of fixed worms was performed as described by Pino *et al*. 26. Worm samples were collected as described in the Sudan Black staining. Animals were washed twice with M9 buffer. After the final wash, worms were fixed in 40% isopropanol at room temperature for 3 min. The fixed worms were stained in Nile red/isopropanol solution for 30 min at room temperature with gentle rocking. The stained worms were washed once with 1 mL M9 buffer and mounted on a 2% agarose pad for microscopy under the fluorescence channel. To compare the fat content in different strains, the pictures were taken with the same camera settings under 1000× magnification as described in the Sudan Black B staining.

### Image quantitation

All quantification was done using fiji/imagej 27. Images were imported as TIFF images, converted to 8‐bit, and then run through Fiji's native threshold algorithm to isolate the lipid droplets. The size of the isolated lipid droplets was then quantified by taking an area measurement immediately posterior to the second bulb of the pharynx. graphpad prism 5 (GraphPad Software, La Jolla, CA, USA) was used to generate column graphs and to perform Student's *t*‐test.

## Results

### *atg‐18* mutations suppress fat accumulation in *daf‐2* mutant dauer larva

**Figure 1:**
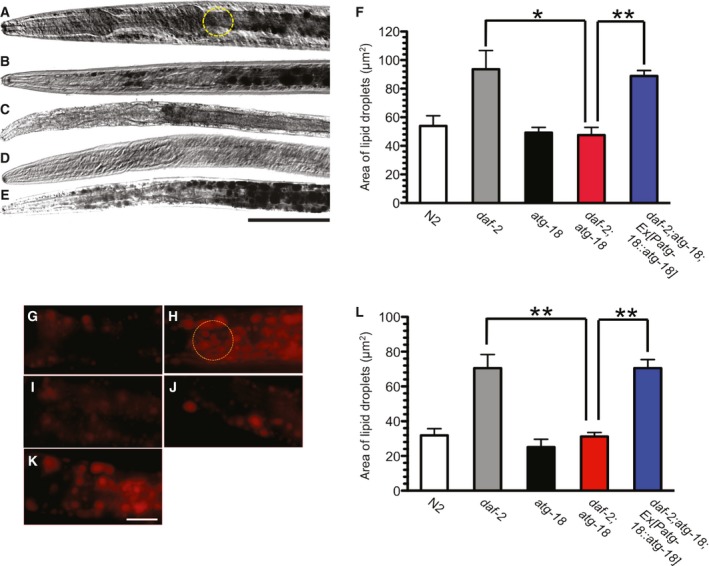
*atg‐18* mutations suppress fat accumulation in *daf‐2* mutant dauer larva. Representative pictures of fat droplets stained by Sudan Black B in wild‐type N2 L3 larva (A), *daf‐2*(*e1370*) dauer (B), *atg‐18*(*gk378*) L3 larva (C), *daf‐2*(*e1370*);*atg‐18*(*gk378*) dauer (D), and *daf‐2*;*atg‐18* dauer carrying natively expressed ATG‐18 (*Patg‐18*::*atg‐18*; E). Scale bar: 50 μm. (F) Quantitation of fat accumulation. Representative pictures of fat droplets stained by Nile red in wild‐type N2 L3 larva (G), *daf‐2* dauer (H), *atg‐18* L3 larva (I), *daf‐2*;*atg‐18* dauer (J), and *daf‐2*;*atg‐18* dauer carrying natively expressed ATG‐18 (*Patg‐18*::*atg‐18*; K). The anterior of the worm body in the image points to the left. Scale bar: 10 μm. (L) Quantitation of fat accumulation. The fat quantitation was performed using fiji/imagej. The size of the isolated lipid droplets was quantified by taking an area measurement immediately posterior to the second bulb of the pharynx, indicated by a dashed, 300‐pixel‐diameter, yellow circle in A for Sudan Black B staining and H for the Nile red staining. Asterisks indicate statistically significant differences relative to controls (* *P* < 0.01, ** *P* < 0.001, *t*‐test). The error bars indicate mean ± standard error of the mean. Total number for each sample: Sudan Black staining, N2, *n* = 13; *daf‐2*,* n* = 6; *atg‐18*,* n* = 6; *daf‐2*;*atg‐18*,* n* = 11; *daf‐2*;*atg‐18*;* Ex*[*Patg‐18*::*atg‐18*], *n* = 34. Nile red staining, N2, *n* = 31; *daf‐2*,* n* = 39; *atg‐18*,* n* = 24; *daf‐2*;*atg‐18*,* n* = 23; *daf‐2*;*atg‐18*;* Ex*[*Patg‐18*::*atg‐18*], *n* = 20.

Sudan Black B was used to stain fat droplets in wild‐type N2, *atg‐18*(*gk378*) mutant, and *daf‐2*(*e1372*) and *daf‐2*(*e1372*);*atg‐18*(*gk378*) mutant dauer larva. The size of the isolated lipid droplets was then quantified by taking an area measurement immediately posterior to the second bulb of the pharynx, indicated by a dashed, yellow circle in Fig. 1A. As reported previously, *daf‐2* dauers significantly increased fat accumulation compared to N2L3 larvae (*P* = 0.0105, *t*‐test; Fig. 1A,B,F). *atg‐18* mutant L3 larvae showed a similar level of fat accumulation to N2 worms (*P* = 0.6794, *t*‐test; Fig. 1C,F). Moreover, the *atg‐18*(*gk378*) mutation significantly suppressed fat accumulation in *daf‐2* dauers (*P* = 0.0016 for *daf‐2* vs. *daf‐2*;*atg‐18* and *P* = 0.8309 for *atg‐18* vs. *daf‐2*;*atg‐18*,* t*‐test; Fig. 1D,F). These data are consistent with the previously published results that mutations of autophagy gene *bec‐1* block fat accumulation in *daf‐2* dauers 14. When a natively expressed *atg‐18* transgene was introduced into *daf‐2*;*atg‐18* mutants, the fat accumulation phenotype of *daf‐2* mutants was restored (*P* < 0.0001 for *daf‐2*;*atg‐18*;*Ex*[*Patg‐18::atg‐18*] vs. *daf‐2*;*atg‐18* and *P* = 0.6551 for *daf‐2*;*atg‐18*;*Ex*[*Patg‐18*::*atg‐18*] vs. *daf‐2*,* t*‐test; Fig. 1E,F), indicating that the decreased fat accumulation in *daf‐2*;*atg‐18* mutants is specifically due to the loss of the *atg‐18* gene.

To confirm the results, we repeated the experiment using another widely used fat staining method: Nile red staining 26. Similar to Sudan Black staining, Nile red also detected increased fat accumulation in *daf‐2* mutant worms compared to N2 (Fig. 1G,H) and showed that *atg‐18* mutations block the fat accumulation in *daf‐2* mutants (Fig. 1I,J,L; *P* < 0.0001 for *daf‐2* vs. *daf‐2*;*atg‐18*). Moreover, natively expressed *atg‐18* transgene restored fat accumulation in *daf‐2*;*atg‐18* mutants (*P* < 0.0001 for *daf‐2*;*atg‐18*;*Ex*[*Patg‐18*::*atg‐18*] vs. *daf‐2*;*atg‐18*,* t*‐test; Fig. 1J,K,L). Thus, *atg‐18* is essential for fat metabolism regulated by the DAF‐2 insulin‐like signaling pathway in dauer larva.

### Tissue‐specific requirement of *atg‐18* for fat metabolism in *daf‐2* mutant dauer larva

**Figure 2:**
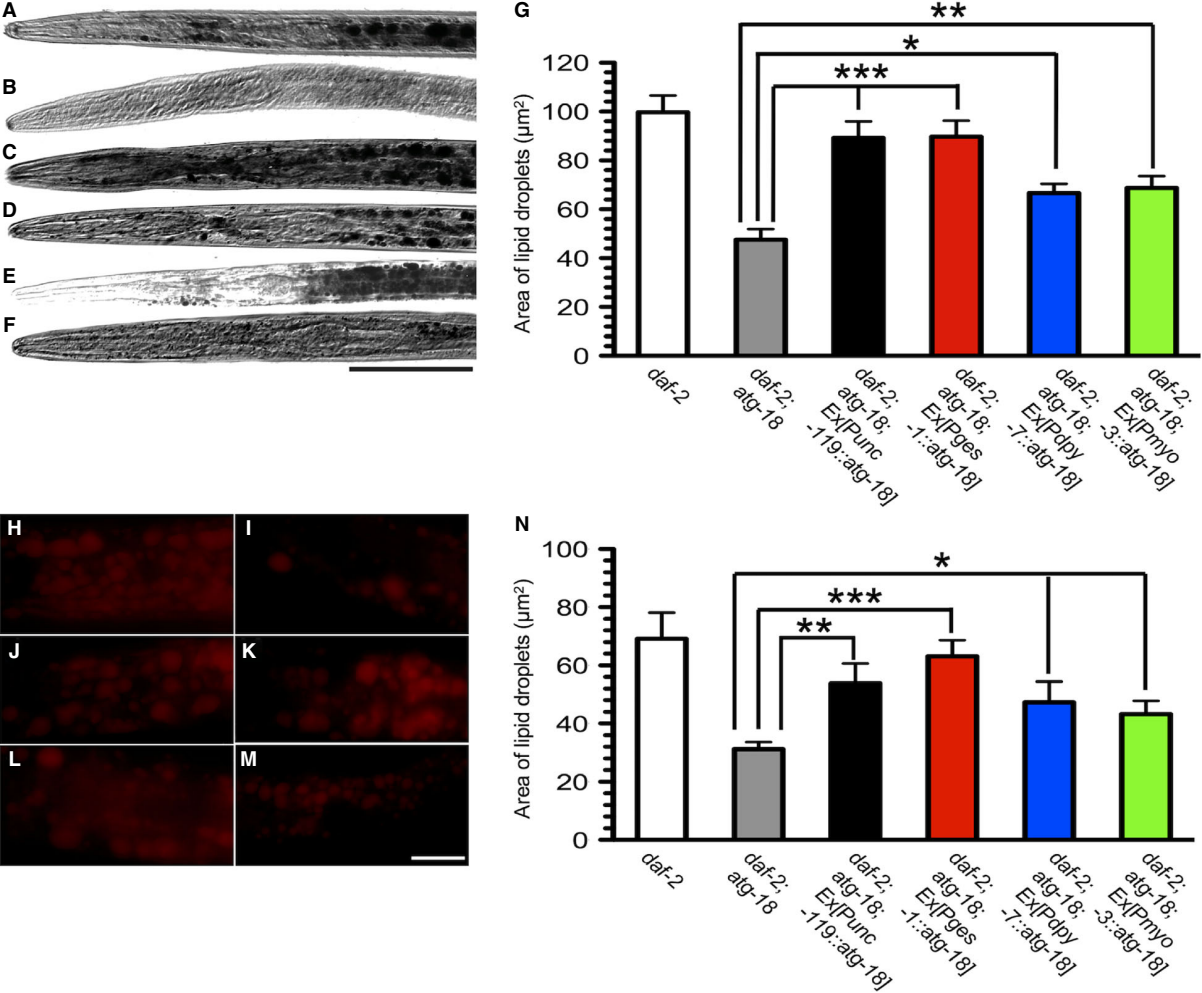
Tissue‐specific requirement of *atg‐18* for fat metabolism in *daf‐2* mutants. Representative pictures of fat accumulation in the indicated strains: (A) *daf‐2*, (B) *daf‐2*;*atg‐18*, (C) *daf‐2*;*atg‐18*;*Ex*[*Punc‐119*::*atg‐18*], (D) *daf‐2*;*atg‐18*;*Ex*[*Pges‐1*::*atg‐18*], (E) *daf‐2*;*atg‐18*;*Ex*[*Pdpy‐7*::*atg‐18*], and (F) *daf‐2*;*atg‐18*;*Ex*[*Pmyo‐3*::*atg‐18*]. Scale bar: 50 μm. (G) Quantitation of fat accumulation in the indicated strains. Representative pictures of fat droplets stained by Nile red in (H) *daf‐2*, (I) *daf‐2*;*atg‐18*, (J) *daf‐2*;*atg‐18*;*Ex*[*Punc‐119*::*atg‐18*], (K) *daf‐2*;*atg‐18*;*Ex*[*Pges‐1*::*atg‐18*], (L) *daf‐2*;*atg‐18*;*Ex*[*Pdpy‐7*::*atg‐18*], and (M) *daf‐2*;*atg‐18*;*Ex*[*Pmyo‐3*::*atg‐18*]. The anterior of the worm body in the image points to the left. Scale bar: 10 μm. (N) Quantitation of fat accumulation. Asterisks indicate statistically significant differences relative to controls (* *P* < 0.05, ** *P* < 0.01, *** *P* ≤ 0.0001, *t*‐test). The error bars indicate mean ± standard error of the mean. Total number for each sample: Sudan Black staining, *daf‐2*,* n* = 6; *daf‐2*;*atg‐18*,* n* = 8; *daf‐2*;*atg‐18*;* Ex*[*Punc‐119*::*atg‐18*], *n* = 10; *daf‐2*;*atg‐18*;* Ex*[*Pges‐1*::*atg‐18*], *n* = 10; *daf‐2*;*atg‐18*;* Ex*[*Pdpy‐7*::*atg‐18*], *n* = 30; *daf‐2*;*atg‐18*;* Ex*[*Pmyo‐3*::*atg‐18*], *n* = 10. Nile red staining, *daf‐2*,* n* = 23; *daf‐2*;*atg‐18*,* n* = 23; *daf‐2*;*atg‐18*;* Ex*[*Punc‐119*::*atg‐18*], *n* = 32; *daf‐2*;*atg‐18*;* Ex*[*Pges‐1*::*atg‐18*], *n* = 25; *daf‐2*;*atg‐18*;* Ex*[*Pdpy‐7*::*atg‐18*], *n* = 25; *daf‐2*;*atg‐18*;* Ex*[*Pmyo‐3*::*atg‐18*], *n* = 22.

To examine the tissues‐specific requirement of *atg‐18* for fat accumulation in *daf‐2* dauers, the *atg‐18* transgene was expressed under the control of different tissue‐specific promoters. Expression of *atg‐18* in neurons (*Punc‐119*) or intestinal cells (*Pges‐1*) significantly increased fat accumulation in *daf‐2*;*atg‐18* (*P* < 0.0001 for *daf‐2*;*atg‐18*;*Ex*[*Punc‐119*::*atg‐18*] vs. *daf‐2*;*atg‐18* and *P* < 0.0001 for *daf‐2*;*atg‐18*;*Ex*[*Pges‐1*::*atg‐18*] vs. *daf‐2*;*atg‐18*,* t*‐test; Fig. 2A–D,G). Statistical analysis of fat storage also showed that expression of *atg‐18* in hypodermis (*Pdpy‐7*) and body wall muscles (*Pmyo‐3*) partially restored fat accumulation in *daf‐2*;*atg‐18* mutants (*P* < 0.05 for *daf‐2*;*atg‐18*;*Ex*[*Pdpy‐7*::*atg‐18*] vs. *daf‐2*;*atg‐18* and *P* < 0.01 for *daf‐2*;*atg‐18*;*Ex*[*Pmyo‐3*::*atg‐18*] vs. *daf‐2*;*atg‐18*; Fig. 2E–G). Nile red staining shows a similar result (Fig. 2H–N). In conclusion, *atg‐18* in neurons and intestine plays a major role in DAF‐2‐regulated fat metabolism in dauer larva.

### Expression of *atg‐18* in chemosensory neurons is vital for fat accumulation in *daf‐2* mutant dauers

**Figure 3:**
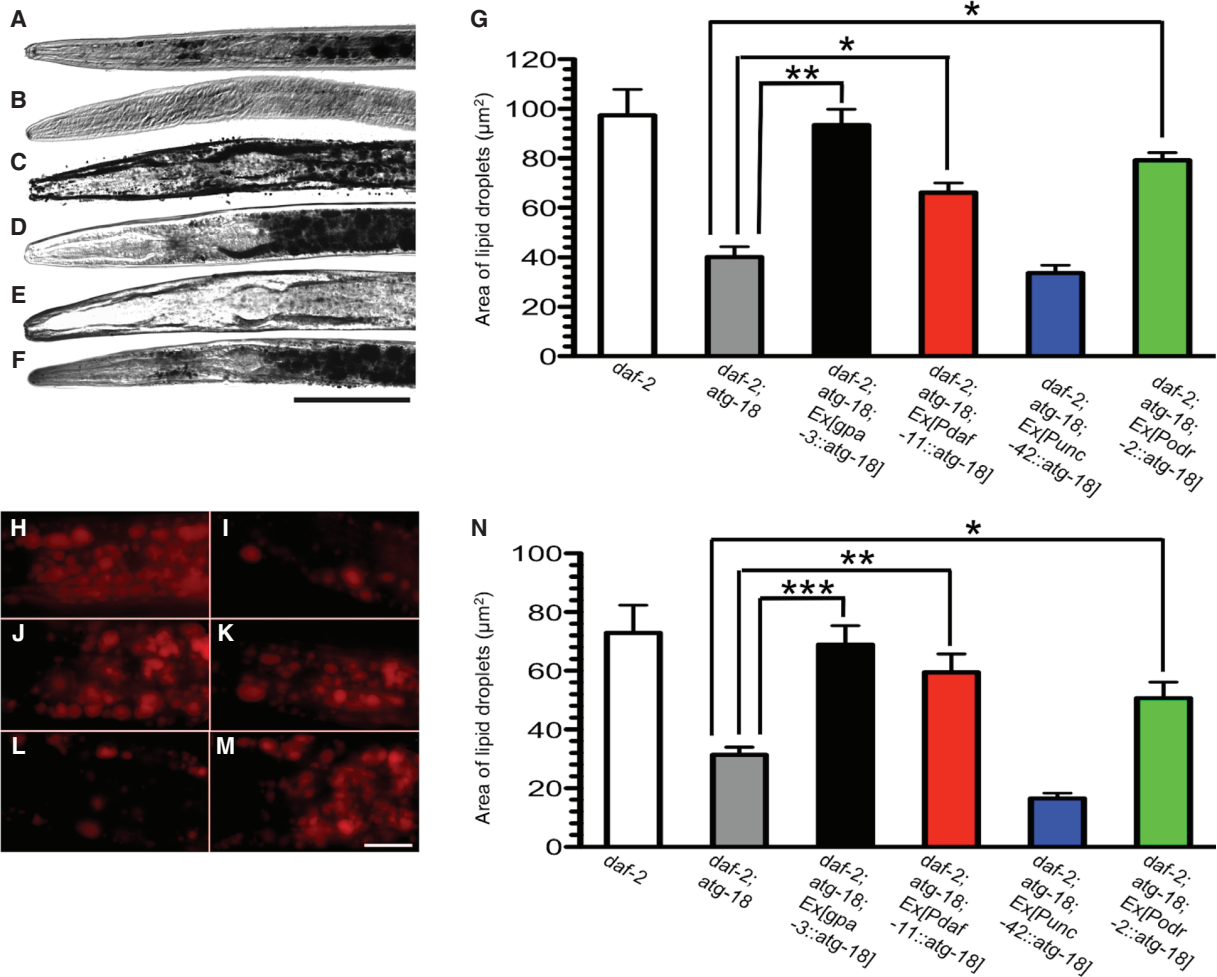
Expression of *atg‐18* in chemosensory neurons is vital for fat accumulation in *daf‐2* mutants. Representative pictures of fat accumulation in (A) *daf‐2*, (B) *daf‐2*;*atg‐18*, (C) *daf‐2*;*atg‐18*;*Ex*[*Pgpa‐3*::*atg‐18*], (D) *daf‐2*;*atg‐18*;*Ex*[*Pdaf‐11*::*atg‐18*], (E) *daf‐2*;*atg‐18*;*Ex*[*Punc‐42*::*atg‐18*], and (F) *daf‐2*;*atg‐18*;*Ex*[*Podr‐2*::*atg‐18*]. Scale bar: 50 μm. (G) Quantitation of fat accumulation. Representative pictures of fat droplets stained by Nile red in (H) *daf‐2*, (I) *daf‐2*;*atg‐18*, (J) *daf‐2*;*atg‐18*;*Ex*[*Pgpa‐3*::*atg‐18*], (K) *daf‐2*;*atg‐18*;*Ex*[*Pdaf‐11*::*atg‐18*], (L) *daf‐2*;*atg‐18*;*Ex*[*Punc‐42*::*atg‐18*], and (M) *daf‐2*;*atg‐18*;*Ex*[*Podr‐2*::*atg‐18*]. Scale bar: 10 μm. (N) Quantitation of fat accumulation. Asterisks indicate statistically significant differences relative to controls (* *P* < 0.05, ** *P* < 0.01, *** *P* ≤ 0.0001, *t*‐test). The error bars indicate mean ± standard error of the mean. Total number for each sample: Sudan Black staining, *daf‐2*,* n* = 6; *daf‐2*;*atg‐18*,* n* = 10; *daf‐2*;*atg‐18*;* Ex*[*Pgpa‐3*::*atg‐18*], *n* = 8; *daf‐2*;*atg‐18*;* Ex*[*Pdaf‐11*::*atg‐18*], *n* = 27; *daf‐2*;*atg‐18*;* Ex*[*Punc‐42*::*atg‐18*], *n* = 22; *daf‐2*;*atg‐18*;* Ex*[*Podr‐2*::*atg‐18*], *n* = 35. Nile red staining, *daf‐2*,* n* = 24; *daf‐2*;*atg‐18*,* n* = 21; *daf‐2*;*atg‐18*;* Ex*[*Pgpa‐3*::*atg‐18*], *n* = 27; *daf‐2*;*atg‐18*;* Ex*[*Pdaf‐11*::*atg‐18*], *n* = 37; *daf‐2*;*atg‐18*;* Ex*[*Punc‐42*::*atg‐18*], *n* = 36; *daf‐2*;*atg‐18*;* Ex*[*Podr‐2*::*atg‐18*], *n* = 37.

We found expression of *atg‐18* gene in ADF, ADL, ASE, ASG, ASH, ASI, ASJ, ASK, AWA, and AWC chemosensory neurons (*Pgpa‐3*::*atg‐18*) restored fat accumulation in *daf‐2*;*atg‐18* (Fig. 3A–C,G). The *atg‐18* gene expressed in ASE, ASI, ASJ, ASK, AWB, and AWC neurons (*Pdaf‐11*::*atg‐18*) also significantly increased fat storage in *daf‐2*;*atg‐18* (*P* = 0.0007 for *daf‐2*;*atg‐18*;*Ex*[*Pdaf‐11*::*atg‐18*] vs. *daf‐2*;*atg‐18*,* t*‐test; Fig. 3D,G). However, expression of *atg‐18* gene in ASH neurons and more than twenty other nonchemosensory neurons (*Punc‐42*::*atg‐18*) did not increase fat storage in *daf‐2*;*atg‐18* mutants (*P* = 0.2366 for *daf‐2*;*atg‐18*;*Ex*[*Punc‐42*::*atg‐18*] vs. *daf‐2*;*atg‐18*,* t*‐test; Fig. 3E,G). These data suggest ATG‐18 in chemosensory neurons except ASH mediates the effect of IGF signaling on fat accumulation in dauer larvae. We recently reported that ATG‐18 in ASG gustatory neurons fully restored *daf‐2*(*e1370*) longevity in *daf‐2*;*atg‐18* worms. We then tested if expression of *atg‐18* gene in ASG neurons plays a similar role for fat metabolism. We found expression of *atg‐18* in ASG neurons (*Podr‐2*::*atg‐18*) significantly increased fat accumulation in *daf‐2*;*atg‐18* mutants (*P* < 0.0001 for *daf‐2*;*atg‐18*;*Ex*[*Podr‐2*::*atg‐18*] vs. *daf‐2*;*atg‐18*,* t*‐test; Fig. 3F,G). We obtained a similar result when we repeated the experiment using the Nile red staining method (Fig. 3H–N). Together, we show that *atg‐18* in chemosensory neurons alone can mediate the effect of DAF‐2 signaling on fat metabolism in dauer larva.

### Neurotransmitters mediate the influence of *atg‐18* on fat metabolism in *daf‐2* mutant dauers

**Figure 4:**
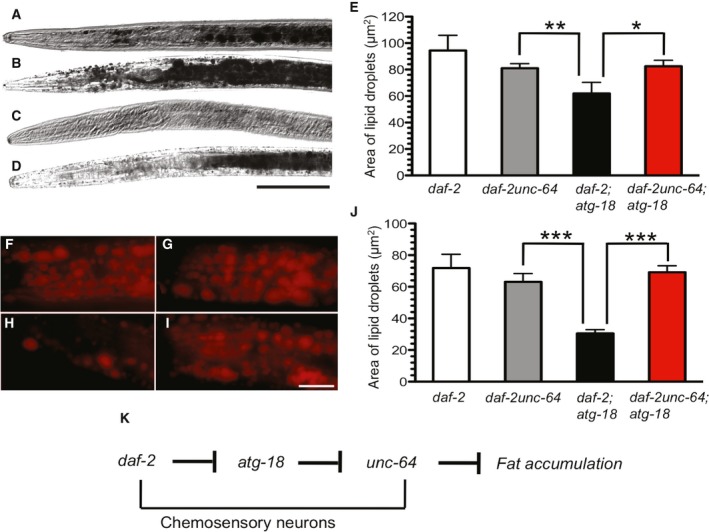
Neurotransmitters mediate the influence of *atg‐18* on fat metabolism in *daf‐2* mutants. Representative pictures of fat droplets in (A) *daf‐2*, (B) *daf‐2unc‐64*, (C) *daf‐2*;*atg‐18,* and (D) *daf‐2unc‐64*;*atg‐18*. Scale bar: 50 μm. (E) Quantitation of fat accumulation. Representative pictures of fat droplets stained by Nile red in (F) *daf‐2*, (G) *daf‐2unc‐64*, (H) *daf‐2*;*atg‐18,* and (I) *daf‐2unc‐64*;*atg‐18*. Scale bar: 10 μm. (J) Quantitation of fat accumulation. Asterisks indicate statistically significant differences relative to controls (* *P* < 0.05, ** *P* < 0.01, ***P ≤ 0.0001, *t*‐test). The error bars indicate mean ± standard error of the mean. Total number for each sample: Sudan Black staining, *daf‐2*,* n* = 6; *daf‐2unc‐64*,* n* = 19; *daf‐2*;*atg‐18*,* n* = 12; *daf‐2unc‐64*;*atg‐18*,* n* = 19. Nile red staining, *daf‐2*,* n* = 24; *daf‐2unc‐64*,* n* = 21; *daf‐2*;*atg‐18*,* n* = 22; *daf‐2unc‐64*;*atg‐18*,* n* = 21. (K) A genetic pathway depicting the role of *atg‐18* in IGF‐regulated fat metabolism in dauer larvae. Based on the epistasis relationships between *daf‐2*,* atg‐18,* and *unc‐64* regarding the fat accumulation phenotype of *daf‐2* mutants, it is proposed that the *atg‐18* gene acts downstream of *daf‐2* but upstream of *unc‐64*. Neurotransmitters released via UNC‐64 from chemosensory neurons inhibit fat accumulation. Wild‐type gene functions are shown with T‐bars indicating inhibition.

We investigated whether release of neurotransmitters is required for ATG‐18 to control fat metabolism cell nonautonomously. Mutations in *unc‐64*, the gene that encodes the worm ortholog of vertebrate syntaxin 1A, block the release of neurotransmitters 28. Figure 4 shows that the *unc‐64*(*e246*) mutation has no statistically significant influence on fat accumulation in *daf‐2* dauers (*P* = 0.1327, *t*‐test; Fig. 4A,B,E). Interestingly, although *daf‐2*;*atg‐18* mutants stored significantly less fat droplets compared to *daf‐2unc‐64* (*P* < 0.001 for *daf‐2unc‐64* vs. *daf‐2*;*atg‐18*,* t*‐test), the triple mutant *daf‐2unc‐64*;*atg‐18* had a similar amount of fat droplets when compared to *daf‐2* (*P* = 0.2529 for *daf‐2* vs. *daf‐2unc‐64*;*atg‐18*,* t*‐test; Fig. 4B–E). These epistasis data suggest that certain neurotransmitters act downstream of ATG‐18 to mediate the influence of IGF signaling on fat metabolism in *C. elegans* dauer larvae. These observations were confirmed by Nile red staining (Fig. 4F–J). Thus, ATG‐18 influences fat metabolism through a neuroendocrine mechanism in *daf‐2* mutant dauer larva.

## Discussion

*Caenorhabditis elegans* has a conserved insulin‐like signaling pathway, and the *daf‐2* gene encodes the single insulin‐like receptor tyrosine kinase. Here, we show that mutations of autophagy gene *atg‐18* completely block fat accumulation in *daf‐2* mutant dauer larva, which is consistent with the previous report that inactivation of autophagy gene *bec‐1* suppresses fat storage in *daf‐2* dauers 14. Moreover, our data indicate that ATG‐18 acts primarily in neurons and intestinal cells to mediate the influence of DAF‐2 signaling on fat metabolism in dauer larvae. By contrast, ATG‐18 in hypodermis and body wall muscles plays a minor role. We recently reported that ATG‐18 in neurons, intestinal cells, and the hypodermis can fully restore the lifespan of *daf‐2*;*atg‐18* mutants to *daf‐2* mutant level, while ATG‐18 in body wall muscles only modestly increases the lifespan of *daf‐2*;*atg‐18* mutants 17. Thus, although neuronal and intestinal ATG‐18 functions similarly in DAF‐2‐regulated lifespan and fat metabolism, hypodermal ATG‐18 has a different role in these two processes. Moreover, ATG‐18 in body wall muscle is not essential for both of these two *daf‐2* mutant phenotypes.

*Caenorhabditis elegans* utilizes chemosensory neurons to detect environmental cues 29. We found that expression of the *atg‐18* gene under the control of *gpa‐3* and *daf‐11,* but not *unc‐42,* promoters significantly increases fat storage in *daf‐2*;*atg‐18* mutants. These data indicate that ATG‐18 in chemosensory neurons except ASH mediates the influence of DAF‐2 signaling on fat metabolism in dauer larvae. Our recent report shows that ATG‐18 in ASE, ASI, ASJ, ASK, AWB, and AWC neurons has no statistically significant influence on DAF‐2‐regulated lifespan extension 17. Thus, ATG‐18 in chemosensory neurons functions differently in regulating fat metabolism in dauer larvae and adult lifespan. Indeed, the longevity phenotype can be uncoupled from fat accumulation in *C. elegans* 11, 12. We reported previously that ATG‐18 in ASG neurons is required for DAF‐2‐regulated longevity. Interestingly, expression of ATG‐18 in only ASG neurons (*Podr‐2*::*atg‐18*) significantly increases fat accumulation in *daf‐2*;*atg‐18* mutants, which suggests ATG‐18 in some chemosensory neurons, such as ASG, can regulate both adult lifespan and fat metabolism in dauer larvae.

Neurons communicate through neurotransmitters. The release of neurotransmitters is blocked by *unc‐64* mutations 28. We found that *unc‐64* mutations have no obvious effect on fat accumulation in *daf‐2* dauer larvae (Fig. 4). However, *unc‐64* is epistatic to *atg‐18,* as *daf‐2unc‐64*;*atg‐18* mutants store a significantly higher amount of fat compared to *daf‐2*;*atg‐18* mutants. The genetic interactions of these genes suggest a model illustrated in Fig. 4K. Essentially, DAF‐2 negatively regulates the autophagy process that, in turn, negatively influences the availability of neurotransmitters that suppress fat accumulation. Autophagy could influence biosynthesis of neurotransmitters, package of neurotransmitters into synapse vesicles, and/or release of these chemicals into the synaptic cleft through UNC‐64. It has been reported that worms deficient in biosynthesis of serotonin accumulate fat, and exogenous administration of 5‐hydroxytryptamine (serotonin) increases fat storage in *C. elegans* 30, 31. These findings suggest that serotonin could be a candidate neurotransmitter that is regulated by autophagy to influence fat metabolism. Interestingly, ASG neurons, where ATG‐18 acts to control fat metabolism, can communicate with other neurons through serotonin 32. Of note, in the present work, we only examine the tissue‐specific role of *atg‐18* in fat metabolism in *daf‐2* mutant dauer larvae. Thus, the role of *atg‐18* in wild‐type worms, in adult animals, and in other developmental stages of *C. elegans* remains to be determined. Nevertheless, our data demonstrate that *atg‐18* in chemosensory neurons can mediate the influence of insulin‐like signaling on fat metabolism in dauer larvae. In mammals, insulin signaling in the central nervous system also controls fat homeostasis. Similar to *daf‐2* mutants, knockout mice without neuronal insulin receptors are obese 33. Thus, it is possible that autophagy is downstream of neuronal insulin signaling in controlling fat metabolism in mammals.

## Conflict of interest

The authors declare no conflict of interest.

## Author contributions

RJ and JZ performed the experiments. RJ and KJ analyzed the data and wrote the manuscript. KJ supervised the experiments.
